# New Pathways for the Skin's Stress Response: The Cholinergic Neuropeptide SLURP-1 Can Activate Mast Cells and Alter Cytokine Production in Mice

**DOI:** 10.3389/fimmu.2021.631881

**Published:** 2021-03-18

**Authors:** Christoph M. Ertle, Frank R. Rommel, Susanne Tumala, Yasuhiro Moriwaki, Jochen Klein, Johannes Kruse, Uwe Gieler, Eva M. J. Peters

**Affiliations:** ^1^Psychoneuroimmunology Laboratory, Clinic for Psychosomatic Medicine and Psychotherapy, Justus-Liebig-University Giessen, Giessen, Germany; ^2^Department of Pharmacology, Keio University Faculty of Pharmacy, Tokyo, Japan; ^3^Department of Pharmacology, Biocenter N260, Goethe University Frankfurt, Frankfurt, Germany; ^4^Clinic for Psychosomatic Medicine and Psychotherapy, Justus-Liebig-University Giessen, Giessen, Germany; ^5^Clinic for Psychosomatic Medicine and Psychotherapy, Philipps University of Marburg, Marburg, Germany; ^6^Department of Dermatology, University Hospital Giessen, Giessen, Germany; ^7^Charité Center 12 for Internal Medicine and Dermatology, Charité - Universitätsmedizin Berlin, Berlin, Germany

**Keywords:** Chrna7 knockout, mast cells, alpha7 nicotinic acetylcholine receptor, cholinergic system, secreted Ly-6/uPAR-related protein 1, hypoxia inducible factor 1 alpha, stress

## Abstract

**Background:** The alpha7 nicotinic acetylcholine receptor (Chrna7) plays an essential anti-inflammatory role in immune homeostasis and was recently found on mast cells (MC). Psychosocial stress can trigger MC hyperactivation and increases pro-inflammatory cytokines in target tissues such as the skin. If the cholinergic system (CS) and Chrna7 ligands play a role in these cascades is largely unknown.

**Objective:** To elucidate the role of the CS in the response to psychosocial stress using a mouse-model for stress-triggered cutaneous inflammatory circuits.

**Methods:** Key CS markers (ACh, Ch, SLURP-1, SLURP-2, Lynx1, Chrm3, Chrna7, Chrna9, ChAT, VAChT, Oct3, AChE, and BChE) in skin and its MC (**s**MC), MC activation, immune parameters (TNFα, IL1β, IL10, TGFβ, HIF1α, and STAT3) and oxidative stress were analyzed in skin from 24 h noise-stressed mice and in cultured MC (**c**MC) from C57BL/6 or Chrna7-Knockout mice.

**Results:** First, Chrna7 and SLURP-1 mRNA were exclusively upregulated in stressed skin. Second, histomorphometry located Chrna7 and SLURP-1 in nerves and **s**MC and demonstrated upregulated contacts and increased Chrna7+ **s**MC in stressed skin, while 5 ng/mL SLURP-1 degranulated **c**MC. Third, IL1β+ **s**MC were high in stressed skin, and while SLURP-1 alone had no significant effect on **c**MC cytokines, it upregulated IL1β in **c**MC from Chrna7-KO and in IL1β-treated wildtype **c**MC. In addition, HIF1α+ **s**MC were high in stressed skin and Chrna7-agonist AR-R 17779 induced ROS in **c**MC while SLURP-1 upregulated TNFα and IL1β in **c**MC when HIF1α was blocked.

**Conclusions:** These data infer that the CS plays a role in the regulation of stress-sensitive inflammatory responses but may have a surprising pro-inflammatory effect in healthy skin, driving IL1β expression if SLURP-1 is involved.

## Introduction

The present concept of how peripheral organs engage in a systemic stress response, describes the upregulation of neuronal and non-neuronal neuroendocrine factors in peripheral tissues as a consequence of perceived stress. Along this so called brain-body-axis, neuroendocrine mediators such as cortisol (nor)adrenaline, substance P (SP), or nerve growth factor (NGF) are generated by the activation of systemic stress response systems such as the hypothalamus-pituitary-adrenal axis (HPA), the sympathetic axis (SA) and the sensory nervous system (SNS). They are then transported into the peripheral organs via blood vessels or reach them via peripheral autonomic and sensory nerve fibers (1). Organs at the self-environment interface such as the skin, lung or gut are densely innervated and have a rich blood supply, which enables a close neuro-immune interaction in these organs in response to systemic stressors. In parallel and interacting with this systemic stress response, there exist neuroendocrine systems of the peripheral organs themselves. Epithelial cells and immunocytes for example produce and secrete neuroendocrine stress mediators locally and communicate via auto- and para-mechanisms with the systemic stress response systems (2–8). In the stress response, this close interaction serves the maintenance of homeostasis but can become psychotoxic if exaggerated (9, 10).

The CS is a highly preserved neuroendocrine signaling system that contributes to allostasis of both non-neuronal and neuronal cells (11–14). Acetylcholine is produced and released by various epithelial and immune cells in response to stress (7, 15, 16), including innate immunocytes such as mast cells (MC), which line organs bordering the environment and which can become activated by stress and its mediators (17, 18). This suggests that this neurotransmitter may also play a role in brain-body interaction. Moreover, ChAT+ nerve fibers can be found to make close contacts with immunocytes in epithelial tissues, which suggests that both the neuronal and non-neuronal cholinergic systems (CS) participate in the CS stress response (11–14).

Recently, psychosocial stress was shown to result in a prominently altered expression of the alpha7 nicotinic acetylcholine receptor (Chrna7) together with an altered expression of a peptidergic modulator of Chrna7 activity, the Secreted Ly-6/uPAR-related protein 1 (SLURP-1) (7).

Chrna7 is known for its impressive capacity to attenuate toxic hyper-inflammatory responses to a wide variety of stressors and it is reduced in chronic inflammatory skin diseases (19–21). This drew our attention to SLURP-1 as a potential controller of pro-inflammatory responses to stress (22). To our surprise, a screen of the respective literature revealed that the precise role of the CS in the response to psychosocial stress is largely unknown and even less is known about a role for SLURP-1. Some information was even contradictory, suggestive of both, a protective and a harmful role of the CS in host defense and inflammatory disease (12, 16, 23–25). This called for research that clarifies the role of SLURP-1 at the crossroads between pro-inflammatory and anti-inflammatory stress responses.

Since prominent SLURP-1 expression was found in the skin, we decided to focus on the skin as a target organ to study SLURP-1 in stress. Placed at the border between self and environment, the skin acts as the first line of defense against environmental challenges and protects the organism from harm (26–30). Innate immune cells such as MC stand in this first line to serve their duty by locally sensing and responding to tissue insults such as bacteria or tumor cells. They do this in close interaction with the skin's innervation that links the skin's stress response to systemic stress cascades that prepare the organism for flight or fight and promote an adaptive response to the stress (31–35). When skin MC are hyperactivated by psychotoxic stress however, they can initiate and worsen inflammation to an extent that makes it maladaptive and promotes allergic inflammation or initiates tissue damage even in otherwise healthy skin (36–41). We had previously shown this in studies focusing on the sensory neuropeptide SP as a key mediator of psychotoxic stress in skin and driver of deleterious inflammation (4, 6, 41, 42).

As SLURP-1 is widely considered to be an allosteric agonist of the Chrna7 expressed by primary sensory neurons, epithelial cells and selected cells of the immune system (43–46), we expected SLURP-1 to have an attenuating effect on these pro-inflammatory cascades. To fill this knowledge gap, we analyzed the skin of mice stressed by noise-exposure and MC from wildtype (WT) and Chrna7-Knockout (Chrna7-KO) mice to answer the following questions: (1) Are SLURP-1 and Chrna7 together with other CS components regulated in the skin by stress and, thus, who are the relevant players in the healthy skin's stress response? (2) What are the neuronal and non-neuronal cellular targets of thus identified stress-sensitive players of the skin's CS? And (3) Does the CS modulate the skin's cytokine-balance of pro- and anti-inflammatory stress responses? The answers to these questions will be highly valuable for the future tailoring of CS interventions designed to treat maladaptive inflammatory stress responses.

## Materials and Methods

### Animals

Twenty female 7–8 weeks old C57BL/6 mice (Charles River, Sulzfeld, Germany) were randomly sorted into two groups (control, stress) on arrival at the animal facility at Universitätsmedizin-Charité, Berlin, Germany as described previously (36). Animal care and experimental procedures of the stress experiments were approved by the institutional review board and conformed to the requirements of the state authority for animal research (LaGetSi, Berlin, Germany) and complied with the ARRIVE guidelines and were carried out in accordance with the EU Directive 2010/63/EU for animal experiments. Additionally, whole thickness skin biopsies with a diameter of 3 mm and both bone marrow-derived and peritoneal MC were obtained for culture (**c**MC) from 20 to 24 weeks old WT and Chrna7-KO mice as described previously (47, 48). The corresponding background strain (C57BL/6J) was used as WT control mice with permission of the state of Hesse, Regierungspräsidium Giessen, according to section 8 of the German Law for the Protection of Animals and conform with the NIH guide for the care and use of laboratory animals. Generation and characterization of these Chrna7-KO animals are described elsewhere (49). All mice were housed under a 12-h-light: 12-h-dark cycle in temperature-regulated rooms (22–24°C), under ambient humidity, with food and water *ad libitum* until experiments were performed. All animals used in this study were genotyped as described by Moser et al. (50). Approximately 0.5 μg DNA was used for qPCR using the Kapa mouse genotyping Kit (Peqlab, Erlangen, Germany) according to published protocols (51).

### Noise-Stress Mouse Model

As previously described, mice within the stress group were exposed to noise (300 Hz tone, emitted at irregular intervals four times per minute; rodent repellent device, Conrad Electronics, Hirschau, Germany) for a single 24 h period (4, 52). Noise is long known to cause a systemic stress response and at the same time alter local skin homeostasis in humans and mice (53, 54). It is therefore frequently used as an experimental stress paradigm to study biomolecular stress responses to psychosocial stress on the organ level.

### Skin Harvesting

Tissue samples from mice stressed as described above as well as unstressed control mice were collected, snap-frozen in liquid nitrogen and stored at −80°C, 48 h after termination of the stress exposure to be used for microarray, high-performance liquid chromatography (HPLC), qPCR and immunohistochemistry (IHC) protocols as described below and published previously (51, 55, 56).

### HPLC

The mouse back skin biopsies of atopic-like dermatitis (AlD)-stress-model were powdered in liquid nitrogen using a ball mill. Around 15 mg of the powder were dissolved in 500 μl of homogenization buffer (150 μl 1 N acetic acid 99% + 850 μl acetone) for the use in HPLC analysis. Choline (Ch) and Acetylcholine (ACh) measurement was performed by HPLC protocols as previously published (57).

### qPCR

As described above, the mouse back skin biopsies were powdered and ~50 mg biopsy powder was used for RNA isolation followed by qPCR of skin biopsies done according to the protocols of our previous study (51). For RNA isolation from peritoneal lavage derived **c**MC, cell lysates were directly dissolved in RLT buffer, without the ball mill step, and continued as described above. Thirty-two microlitre, containing 4 μg of isolated RNA, were then transcripted into cDNA which was used for qPCR. Specific forward, reverse primers and Taqman probes (TIB MOLBIOL, Berlin, GER) used in this study are described in Table 1. Hypoxanthine Guanine Phosphoribosyltransferase (HPRT) and TATA box binding protein (TBP) were validated as reference genes for qPCR analyses. TBP levels showed best results and were constant between treatment groups as reported by others (58). Therefore, gene expressions were normalized to the internal control TBP using a modified version of ΔΔCT method (59). The means of control animal samples were set to 1.

**Table 1:** List of primers and Taqman probes used for qPCR.

**Genes**	**Primer and probe sequences**
AChE	Forward: CCTGAACCTGAAGCCCTTAGA Reverse: CAGAGTATCGGTGGCGCTG Taqman probe: 6FAM-AAAGCGATTCCAGAAGGCGCA- -BBQ
BChE	Forward: GCTTACCTCTGGGAAGAAGAGTTAA Reverse: TCAGTACTTGTGAAGACAGGCCAC Taqman probe: 6FAM-AGTCGATCCATAATGAAAACTTGGGCAAA- -BBQ
ChAT	Forward: CCAggACggTCCTCTTAAAAgAC Reverse: CCCTgTgTgTgTCACTgAggT Taqman probe: 6FAM-CgggACTCCCTggACATgATCgAgC- -BBQ
Chrm3	Forward: GAGTGAACCATATCCTTTCCCATCA Reverse: CTTGGTCACTTGGTCAGAACG Taqman probe: 6FAM-CACCACAGAGACTCTCCTCTTGAAGTGCT- -BBQ
Chrna7Exon 1-4	Forward: CCTGCAAGGCGAGTTCC Reverse: CTCAGGGAGAAGTACACGGTGA Taqman probe: 6FAM-CTGGTCAAGAACTACAACCCGCTGGA- -BBQ
Chrna7Exon 5-7	Forward: AACGTCTTGGTGAATGCATCTG Reverse: CACCCTCCATAGGACCAGGAC Taqman probe: 6FAM-CATTGCCAGTATCTCCCTCCAGGCA- -BBQ
Chrna7Exon 8-10	Forward: GCCCTTGATAGCACAGTACTTCG Reverse: GATCCTGGTCCACTTAGGCATTT Taqman probe: 6FAM-CAGTGGTCGTGACAGTGATTGTGCTGC- -BBQ
Chrna9	Forward: TACAACAAGGCCGATGACGAG Reverse: TGGTGAGTCCCAGGTGATGAG Taqman probe: 6FAM-CCAACGTGGTCCTGCGGTACGAT- -BBQ
HIF1α	Forward: GGGCCATATTCATGTCTATGATACC Reverse: GGCTCATAACCCATCAACTCAGTAAT Taqman probe: 6FAM-ACGGATGAGGAATGGGTTCACAAATC- -BBQ
IL10	Forward: gACTTTCTTTCAAACAAAggACC Reverse: gCTTggCAACCCAAgTAA Taqman probe: 6FAM-ACTgCTAACCgACTCCTTAATgCAgg- -BBQ
IL1β	Forward: AAAgAATCTATACCTgTCCTgTgT Reverse: gCTTgggATCCACACTCTCC Taqman probe: 6FAM-AAgACggCACACCCACCCTgCAgCT- -BBQ
Lynx1	Forward: CCTGTGGCCCAGGCTCT Reverse: GGTGAAGTAAGTTCGTGTGGTCATAC Taqman probe: 6FAM-TGTGCCTACAATGGAGACAACTGCTTCAA- -BBQ
Oct3	Forward: CTGGCTGATCACCCGGA Reverse: AACAGGATGGGTTACTGACTTCTTCA Taqman probe: 6FAM-AGGTGTTTTCCATTGCACTTAGCCACGC- -BBQ
SLURP-1	Forward: gAgCATgggCTATggTgAgg Reverse: TTgAAggggAACgCTgCTT Taqman probe: 6FAM-TgCAAgATggAAgACACAgCCTgTAAgAC- -BBQ
SLURP-2	Forward: TCATCATTGCCACCCGTTCT Reverse: CCAAGCTGGAGACATCAGGAC Taqman probe: 6FAM-TAGCACATCTTCGTCACCAGAGGCAGA- -BBQ
STAT3	Forward: GCTGGTCAAATTTCCTGAGTTGAA
	Reverse: AGAATGTTAAATTTCCGAGACCCTC Taqman probe: 6FAM-AGCAACATCCCCAGAGTCTTTATCAATGCA- -BBQ
TBP	Forward: gTgAATCTTggCTgTAAACTTgACCT Reverse: gCAgTTgTCCgTggCTC Taqman probe: 6FAM-AAATgCTgAATATAATCCCAAgCgATTTgC- -BBQ
TGFβ2	Forward: GCAAAACCCCAAAGCCAG Reverse: GTGGGAGATGTTAAGTCTTTGGA Taqman probe: 6FAM-TCAATCCGCTGCTCGGCCA- -BBQ
TNFα	Forward: gCCTATgTCTCAgCCTCTTCTCATT Reverse: CCACTTggTggTTTgCTACgA Taqman probe: 6FAM-CCATAgAACTgATgAgAgggAggCCATTT- -BBQ
VAChT	Forward: CCCTTAAgCgggCCTTTC Reverse: CgAAgAgCgTggCATAgTCT Taqman probe: 6FAM-TTgATCgCATgAgCTACgACgTgC- -BBQ

*AChE, acetylcholineesterase; BChE, butyrylcholinesterase; ChAT, cholineacetyltransferase; Chrm3, muscarinic acetylcholine receptor 3; Chrna, alpha nicotinic acetylcholine receptor; HIF, hypoxia inducible factor; IL, interleukin; Lynx1, Ly6/neurotoxin 1; Oct3, organic cation transporter 3; SLURP, secreted Ly-6/uPAR-related protein; STAT3, signal transducer and activator of transcription 3; TBP, TATA box binding protein; TGF, transforming growth factor; TNF, tumor necrosis factor; VAChT, vesicular acetylcholine transporter*.

### IHC

IHC was performed with 14 μm thick mouse back-skin cryostat sections as described previously (51). The primary antibodies used in this study are given in Table 2. Standard controls were performed by omission of the primary antibodies and by incubation with mouse IgG1 instead of primary antibodies. Of the two different antibodies tested for SLURP-1, only the established antibody from Moriwaki et al. gave positive staining (44). No labeling was observed in negative controls of Chrna7 staining as published previously (51). Besides, the Chrna7 antibody was tested on KO tissue and no staining was observed (not shown). Five percentage normal serum (Dianova, Hamburg, Germany) of the species in which the secondary antibody was generated was used as blocking solution. 0.3% Triton X-100 (Sigma-Aldrich, München, Germany) was added to the primary antibodies as detergent. Cell nuclei were counterstained with DAPI (4′,6-Diamidin-2-phenylindol), skin MC (**s**MC) with Fluorescein Avidin D (FITC-Avidin) as described previously (6, 68). ProLong® Gold (Life Technologies, Darmstadt, Deutschland) was used as mounting medium. At least 10 microscopic fields and two sections per animal and antibody were analyzed with a fluorescence microscope (DMI 6000B, Leica, Wetzlar, Germany) and histomorphometry (HM) was performed as described previously (56).

**Table 2:** List of primary and secondary antibodies used for immunofluorescence.

**Target**	**Source**	**Dilution**	**Type/Reference**
Rabbit polyclonal anti TNFα	Abcam (Cambridge, UK)	1:200	ab34674 (60)
Rabbit polyclonal anti IL1β	Abcam (Cambridge, UK)	1:100	ab9722 (61)
Rabbit polyclonal anti IL10	Abbiotec (San Diego, CA, USA)	1:100	250713 (62)
Rabbit polyclonal anti TGFβ	Santa Cruz (Dallas, TX, USA)	1:250	sc-90 (63)
Goat polyclonal anti ChAT	AbD Serotec (Kidlington, UK)	1:200	2080-0000 (64)
Rabbit antiserum SLURP-1	Yasuhiro Moriwaki (Tokyo, JPN)	1:200	(44)
Goat polyclonal anti Chrna7	Abcam (Cambridge, UK)	1:100	ab110851 (65)
Rabbit polyclonal anti HIF1α	Novus biologicals (Centennial, CO, USA)	1:400	NB100-479 (66)
Cy3-conjugated F(ab')_2_ fragment donkey anti-rabbit IgG, polyclonal	Dianova (Hamburg, Germany)	1:200	711-166-152 (67)
Cy3-conjugated F(ab')_2_ fragment donkey anti-goat IgG, polyclonal	Dianova (Hamburg, Germany)	1:200	705-166-147 (67)
FITC-Avidin	Vector laboratories (Burlingame, CA, USA)	1:5000	(68)

### Generation of MC Cultures

Since MC are highly fragile cells and do not survive cell sorting protocols with high cell yields and are generally not amenable to culture after sorting due to poor viability and susceptibility to contamination, we employed the following harvesting, enrichment and amplification method to obtain MC for cell culture experiments: (1) **c**MC from bone marrow. This method yields more immature **c**MC that can be cultured over longer periods of time. (2) **c**MC from peritoneal lavage. This method yields more mature **c**MC. In order to generate MC cultures, standard operating procedures employing growth-factor induction of **c**MC differentiation were followed as published elsewhere (69) with minor modifications as described below.

### Bone Marrow-Derived cMC

Mice were deeply anesthetized with 20 μl per gram body weight of a Ketamin (25 mg/ml) and Xylazin (2%) mixture and killed by cervical dislocation. After cleaning the legs with 70% ethanol and removing them, femur and tibia were exposed from surrounding tissue. Using a sterile syringe, the bones were rinsed with 3 ml of preheated **c**MC medium. The bone marrow was collected and centrifuged at 1,200 rpm for 3 min at room temperature. The supernatant was removed, cells were resuspended in fetal calf serum [FCS] and 10% DMSO and stored in −80°C. Prior to experiments, cells were defrosted, washed and resuspended with **c**MC medium (RPMI medium containing: 10% FCS, 1 M HEPES, 1% non-essential amino acids, 1% penicillin-streptomycin [Pen/strep], 1% glutamine, 1% sodium pyruvate, 20 ng/ml interleukin 3 [IL3] and stem cell factor [SCF]) to a final dilution of 500 000 cells/ml to generate **c**MC. Cells were cultured at 37°C and 5% CO_2._ After 2 days, medium was added according to the cell density. Medium was changed and adapted to cell density once per week thereafter.

### Peritoneal Lavage Derived cMC

On the day of harvesting, mice were deeply anesthetized and killed as described above. Abdominal skin was cleaned with 70% ethanol and an incision was made into the ventral skin, paying great attention to preserve an intact peritoneum. Using a sterile syringe, 5 ml of air and 5 ml of NaCl were injected into the peritoneal cavity. Mice were then gently shaken for at least 5 min and the peritoneal lavage was collected with a fresh syringe and centrifuged at 1,000 rpm for 10 min at 4°C. The supernatant was removed and cells were resuspended with **c**MC medium (RPMI medium containing: 10% fetal calf serum [FCS], 1% penicillin-streptomycin [Pen/strep], 0.001% α-Monothioglycerol [MTG]) to a final dilution of 500,000 cells/ml. **c**MC were cultured at 37°C and 5% CO_2_ with regular medium additions or changes as published previously (70).

### Verification of cMC Generation

**Figure 1:**
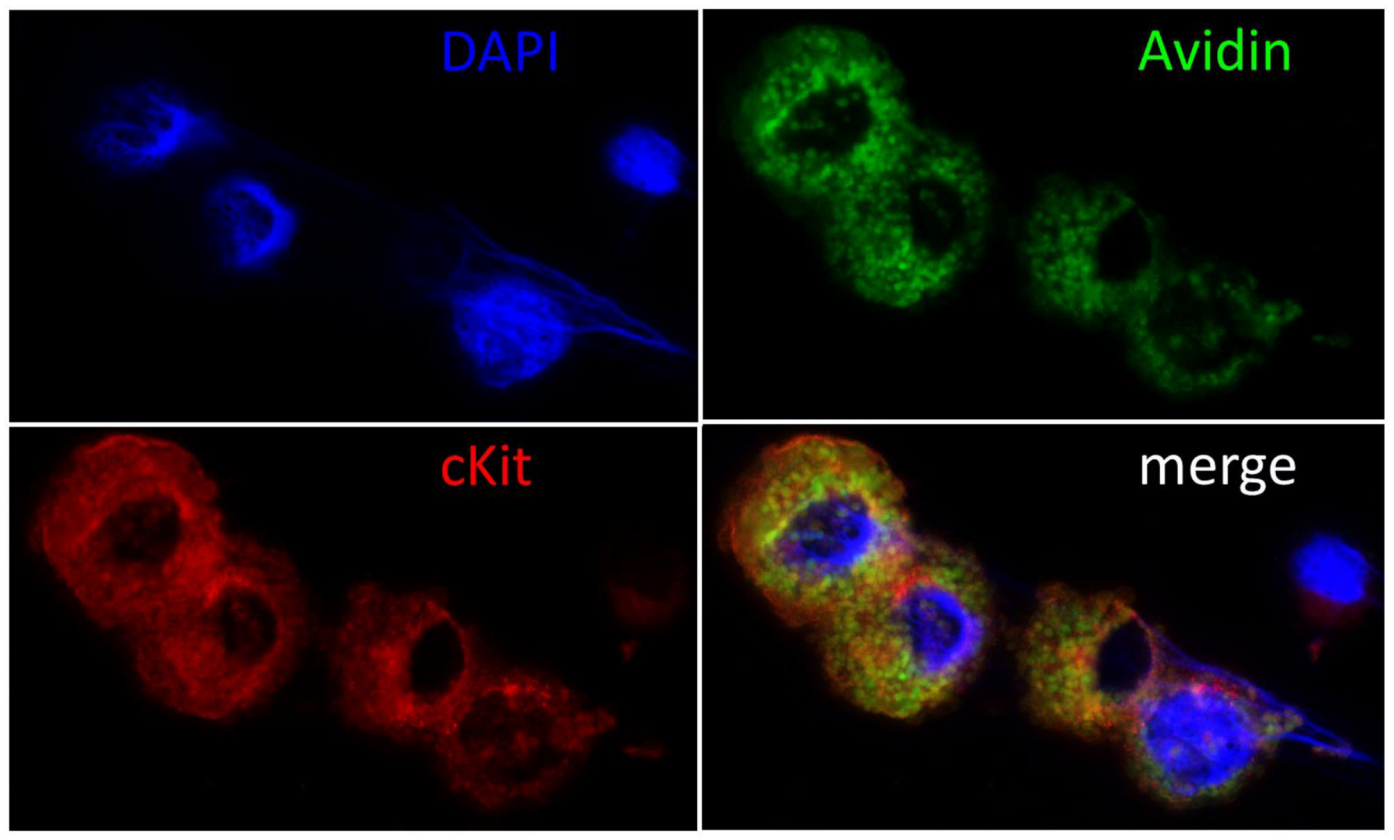
Immunohistochemical confirmation of cMC. Cell nuclei were counterstained blue with DAPI (4′,6-Diamidin-2-phenylindol), MC green with Fluorescein Avidin D (FITC-Avidin) and red with c-Kit staining as described previously (6, 68). Note the large cell nuclei and the intact cytoplasm packed with granules in the double reactive **c**MC and the small nucleus and narrow rim of cytoplasm surrounding the unstained cell, who's morphology suggests it to be a lymphocyte. The majority of c-Kit negative cells showed this morphology prior to cell culture and were lost in passaging.

To verify **c**MC identity and degree of purity of cell cultures, **c**MC cytospins were prepared from both bone marrow and peritoneal lavage originating cell cultures. Cytospins were submitted to Giemsa staining and microscopic analysis of cell reactivity and morphology was performed to identify **c**MC. In addition to the Giemsa staining, **c**MC presence was also verified by **c**MC cytospin staining for the MC specific receptor CD117 in double staining with the unspecific MC marker FITC-Avidin (Figure 1). Staining yield was quantified with the help of an ocular grid, counting stained cells in a minimum of 10 microscopic fields per cytospin. On average, we counted 80.40% **c**MC.

### β-Hexosaminidase Degranulation Assay

**c**MC degranulation was assessed in bone marrow derived **c**MC after 4–6 weeks of culture. They were first sensitized with 1 μg/ml anti-DNP IgE for 1 h. For activation, 100.000 cells/Eppendorf tube were challenged with different stressors and stimulators for 30 min as detailed in Table 3. After that, 50 μL of supernatant or cell lysate and 50 μL of p-NAG (1 mM 4-Nitrophenyl N-acetyl-β-D-glucosaminide in 0.1M Sodium Citrate buffer, pH 4.5) were added to each well of a flat bottom 96-well plate. Color was developed for 90 min at 37°C. The reaction was stopped by adding 100 μL of Sodium Carbonate buffer (50 mM Na_2_CO_3_/NaHCO_3_, pH 10.7). Finally, the absorbance at 405 nm was measured in a microplate reader (TriStar LB 941 Multimode Microplate Reader) both in the supernatant and in the cell lysate.

**Table 3:** Stimulants used in the β-hexosaminidase degranulation assay (HDA).

**Stimulant**	**Concentration**	**Incubation time**	**Company**
SLURP-1	0,5 ng/ml; 5 ng/ml	30 min	Abnova (Heidelberg, GER)
Substance P	10 μM	30 min	Sigma-Aldrich (München, GER)
Nerve growth factor (NGF)	50 ng/ml	30 min	Promega (Madison, WI, USA)
Albumin, dinitrophenyl, (DNP-HSA)	30 ng/ml	30 min	Sigma-Aldrich (München, GER)
Ionomycin	1 μM	30 min	Sigma-Aldrich (München, GER)
Phorbol 12-myristate 13-acetate (PMA)	10 nM	30 min co-incubation with Ionomycin	Sigma-Aldrich (München, GER)

Cells were resuspended and washed in a HEPES-Tyrodes buffer (1,000 ml containing: 135 mM NaCl, 5 mM KCl, 1 mM MgCl_2_, 1.8 mM CaCl_2_, 5.6 mM glucose, 20 mM HEPES, and 0.05% BSA) and lysed through freezing them in −80°C for 15 min. The percentage of the β-hexosaminidase release was calculated using the following formula:

$$\begin{array}{l} \%\beta -\text{hexosaminidase release}=\text{absorption supernatant}/\\ \;\left(\text{absorption supernatant }+\text{ absorption lysed cells}\right) \end{array}$$

Doses for stimulant usage in the respective experiments were either determined by pre-tests or taken from literature. SLURP-1 doses of 0.5 ng/ml, 5 and 50 ng/ml were tested and yealded the best reproducible effects on **c**MC with 5 ng/ml of SLURP-1 while 0.5 ng/ml had very little effect and 50 ng/ml had toxic effects (not shown). SP doses were used according to literature (71). The NGF dose was chosen according to manufacturer's recommendations and the literature (72). Stimulation time of 30 min was chosen according to literature (71).

### cMC Culture With Stimulators of Cytokine Production

On day 11 in culture, peritoneal lavage derived **c**MC were seeded in 6-wells plates at a density of 100,000 cells/100 μl and treated with different stressors and stimulators for 2, 8, 24, and 32 h as detailed in Table 4. After treatment, cells were collected for RNA isolation and supernatants were collected for ELISA analysis. All materials were stored at −80°C until further analysis. Cells were also utilized for **c**MC identification using cytospin followed by Giemsa and c-Kit staining.

**Table 4:** Stressors and stimulators used in cMC cell culture.

**Stimulants**	**Concentrations**	**Incubation times**	**Company**
SLURP-1	5 ng/ml	2, 8, 24 h 8 h pre-incubation Prior to 24 h IL1β	Abnova (Heidelberg, GER)
AR-R 17779 hydrochloride (Chrna7 agonist)	10 μM	2, 8, 24, 32 h 8 h pre-incubation Prior to 24 h SLURP-1	TOCRIS (Bristol, UK)
IL1β	5 ng/ml	24 h	PeproTech (Hamburg, GER)
PX-478 2HCl (HIF1α inhibitor)	40 μM	8 h pre-incubation Prior to 24 h SLURP-1	Selleckchem (Boston, US)

The doses of AR-R 17779 hydrochloride and IL1β were taken from literature (73, 74). The dose of the HIF1α inhibitor PX-478 2HCl was chosen according to manufacturer's information.

### ELISA

Supernatant culture medium of peritoneal lavage derived **c**MC was utilized for HIF1α ELISA (IBL International, Hamburg, Germany) and were performed according to the manufacturer's protocol.

### Microarray Analysis

RNA isolated from the mouse back skin biopsies of stressed and unstressed animals was utilized for microarray analysis as described previously (6).

### Dichlorofluorescein Diacetate (DCFH-DA) Assay

Reactive oxygen species (ROS) generation was assessed by performing a DCFH-DA assay using bone marrow derived **c**MC. **c**MC were first washed and resuspended in the HEPES-Tyrodes buffer (buffer without FCS, phenol red and antibiotics) to a final dilution of 100,000 cells/100 μl. Cells were seeded in a black flat bottom 96-well plate and treated with different stressors and stimulators for 30 min as detailed in Table 5. After 30 min, 20 μM DCFH-DA (Sigma-Aldrich, München, Germany) was added and cells were incubated for another 30 min at 37°C in the dark. Afterwards, cells were washed and DCFH-DA fluorescence was measured at 1, 3, 5, and 24 h after DCFH-DA adding, using a fluorescence microplate reader (Mithras LB 940 Multimode Microplate Reader) with excitation and emission of 485 nm and 535 nm.

**Table 5:** Stimulants used in the DCFH-DA assay.

**Stimulants**	**Concentrations**	**Incubation times**	**Company**
AR-R 17779 hydrochloride (Chrna7 agonist)	10 μM	30 min	TOCRIS (Bristol, UK)
Substance P	10 μM	30 min	Sigma-Aldrich (München, GER)

### Statistics

To fulfill the R principles for the harmonization of science and ethics in the field of animal experimentation, we aimed to keep the number of mice required for the experiments as low as possible. Based on our previous experiences with exploratory animal studies, we presumed that the assumption of normality may not be fulfilled during the estimation of sample size and non-parametric methods were chosen. Alternative hypotheses were tested as two-sided tests for equality with a significance level of 0.05, and the power was calculated by G*Power 3.1 as for example described in (75). This resulted in an *N* = 5 for a small effect sizes of 0.2 and *N* = 3 for 0.1. Therefor all experiments were performed with an *N* = 3–5 per group as indicated in the figures.

GraphPad Prism 6 software (GraphPad Software: La Jolla, US) was used for all other data processing. After testing for normality with the Shapiro-Wilk test, statistical differences between the averages of the control and the stress treated groups and between groups in **c**MC culture experiments were determined non-parametrically by Mann-Whitney *U*-test if only two groups were involved or Kruskal-Wallis test with post hock pairwise comparisons using Dunn's multiple comparisons tests if more than one group was involved. Means and SEM are shown, *p* ≤ 0.05 was interpreted as significant. HM and mRNA data were submitted to a Spearman's rank correlation (Spearman's rho).

## Results

### Noise Stress Exposure Increases Cutaneous Chrna7 and SLURP-1 mRNA

**Figure 2:**
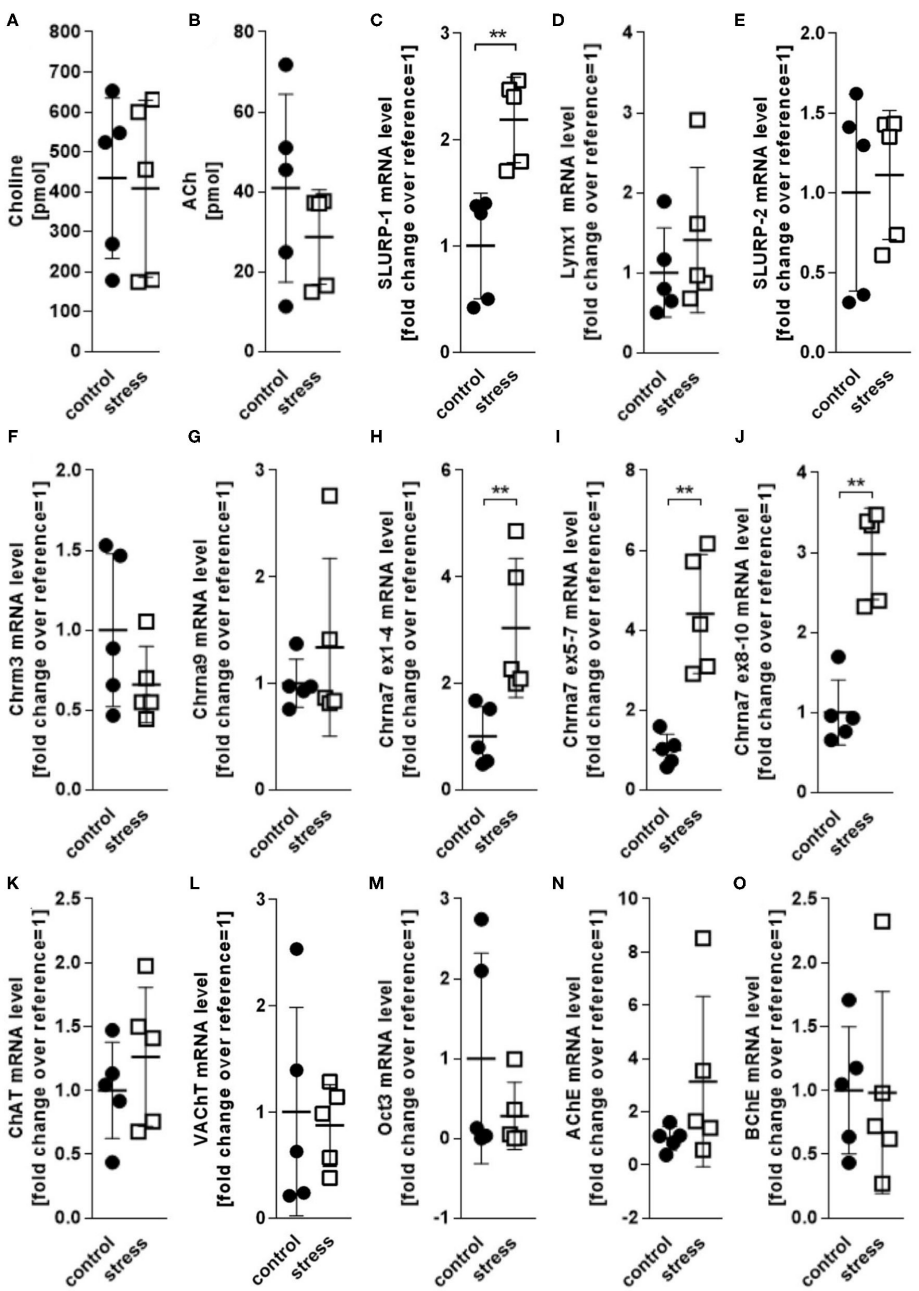
HPLC and qPCR levels of key CS markers detected in full thickness skin samples obtained from control and 24 h noise-stressed mice. *P* < 0.001 = **. Note, that Chrna7 was analyzed with three different primers, targeting exons 1–4, 5–7, and 8–10, respectively, to assess different expression patterns of possible splice variants (51). All three primers yielded the same results. Each dot in the graphs represents one mouse. ACh, acetylcholine; AChE, acetylcholineesterase; BChE, butyrylcholinesterase; ChAT, cholineacetyltransferase; Chrm3, muscarinic acetylcholine receptor 3; Chrna, alpha nicotinic acetylcholine receptor; Lynx1, Ly6/neurotoxin 1; Oct3, organic cation transporter 3; SLURP, secreted Ly-6/uPAR-related protein; VAChT, vesicular acetylcholine transporter.

Reportedly, adverse psychotoxic noise stress effects are prominent in skin 48 h after termination of a 24 h noise-stress exposure (4, 6, 36). We analyzed cholinergic parameters in full thickness skin samples from mice exposed to 24 h of noise stress 48 h after stress exposure (Figure 2). We found that the levels of ACh and choline as measured in skin extracts were unchanged (Figures 2A,B). Accordingly, the mRNA expression of presynaptic cholinergic markers responsible for ACh and choline synthesis (ChAT, VAChT, Oct3) were unchanged after noise stress, as was the expression of digesting cholinesterases (AChE and BChE) (Figures 2C–O). However, the mRNA levels of SLURP-1 and Chrna7 were significantly upregulated by noise-stress exposure (Figures 2C,H–J). No other receptor subtypes (Chrna9, Chrm3) and Chrna modulators (Lynx1, SLURP-2) were changed.

### Nerve Fibers and MC Are Source and Target of SLURP-1 and Chrna7

#### SLURP-1 and Chrna7 Are Expressed at the sMC-Nerve Fiber Interface

**Figure 3:**
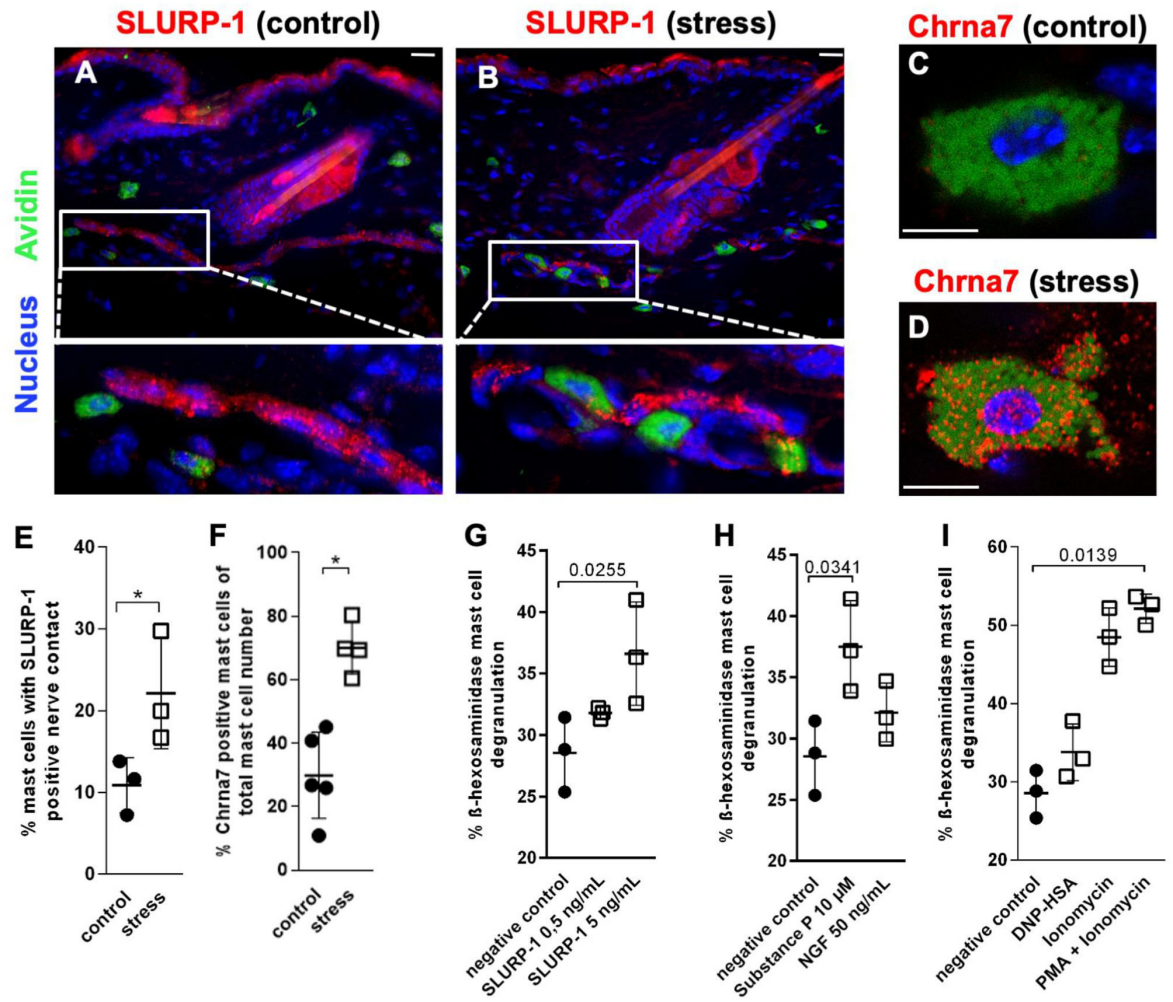
MCs are targets of SLURP-1 in the skin. **(A–D)** immunohistology and **(E,F)** HM of Chrna7 and SLURP-1 in full-thickness skin samples obtained from control and 24 h noise-stressed mice. In photomicrographs, **s**MC are labeled green, Chrna7 and SLURP-1 red. Each dot in the graphs represents one mouse. **(G–I) c**MC stimulation with SLURP-1 yielded comparable results to stimulation with the stress mediator SP and standard MC degranulators DNP-HSA, Ionomycin, Ionomycin and PMA. *P* < 0.01 = *. Scale bars: **(A,B)** 25 μm and **(C,D)** 10 μm. Chrna, alpha nicotinic acetylcholine receptor; DNP-HSA, Dinitrophenyl Albumin; NGF, Nerve Growth Factor; PMA, Phorbol 12-myristate 13-acetate; SLURP, secreted Ly-6/uPAR-related protein.

To determine the skin's target structures for stress-induced CS regulation of Chrna7 and its endogenous peptidergic ligand SLURP-1, we next employed IHC of full thickness skin samples cryo-conserved 48 h after a 24 h noise-stress exposure. This approach allows the study of psychotoxic stress effects *in situ*. Full thickness tissue samples taken from stressed experimental animals are best suited to show the stress-responses of the cells, while they are embedded in their connective tissue and in their natural vascular and neuronal environment. This approach revealed prominent expression of SLURP-1 and Chrna7 in nerve fibers and **s**MC and suggested they are source and target of CS-stress effects in healthy skin (Figures 3A–D). In addition, a weak expression of SLURP-1 was found in the basal layer of the epidermis, but immunoreactivity of other immune cells, including the suprabasally located Langerhans cells or dermal macrophages and lymphocytes, could not be detected.

SLURP-1-immunoreactive nerve fibers showed the characteristic beads-on-a-string like morphology of peptidergic nerve fibers, as described previously with neuronal markers like PGP 9.5 and neuropeptides such as SP (44, 55, 56, 76). They were frequently found in the subcutis, but also abundant subepidermally, in the innervation of arrector pili muscles and around hair follicles. Close contacts between SLURP-1+ nerve fibers and **s**MC were most prominent in stressed mice at the dermis-subcutis border and often looked as if the nerve fibers were embracing the **s**MC. Also primarily in stressed mice, Chrna7+ **s**MC showed a granular cytoplasmatic staining pattern indicating a vesicular staining (Figures 3C,D). This suggested increased neuro-immune interaction between SLURP-1 peptidergic nerve fibers and MC in stressed mice.

HM was used for the quantification of these nerve fiber contacts with MC and MC-Chrna7 expression. This approach confirmed significantly more contacts between SLURP-1+ nerve fibers and **s**MC in the skin of noise-stressed mice (Figure 3E). At the same time, the percentage of Chrna7+ **s**MC was increased after stress exposure (Figure 3F), but the percentage of ChAT+ **s**MC or the total number of **s**MC was unchanged (data not shown).

### SLURP-1 Degranulates cMC

Corresponding to the frequent SLURP-1+ nerve fiber contacts with **s**MC in stressed skin, higher doses of SLURP-1 (5 ng/ml) degranulated **c**MC, whereby SLURP-1 had an effect comparable to the classical stress-MC degranulator SP and stronger than NGF, but was less capable to induce degranulation than the combination of the potent MC degranulators PMA and Ionomycin (Figures 3G–I). This adds SLURP-1 to the list of peptides that act as stress-MC degranulators. As it is localized in peripheral nerve fibers with peptidergic morphology and modulates the function of a neurotransmitter receptor it may be considered as a neuropeptide in this context.

### Pro-inflammatory MC Cytokines Are CS Regulated

#### IL1β sMC Expression Is Enhanced by Stress and by SLURP-1 Under Inflammatory Conditions

**Figure 4:**
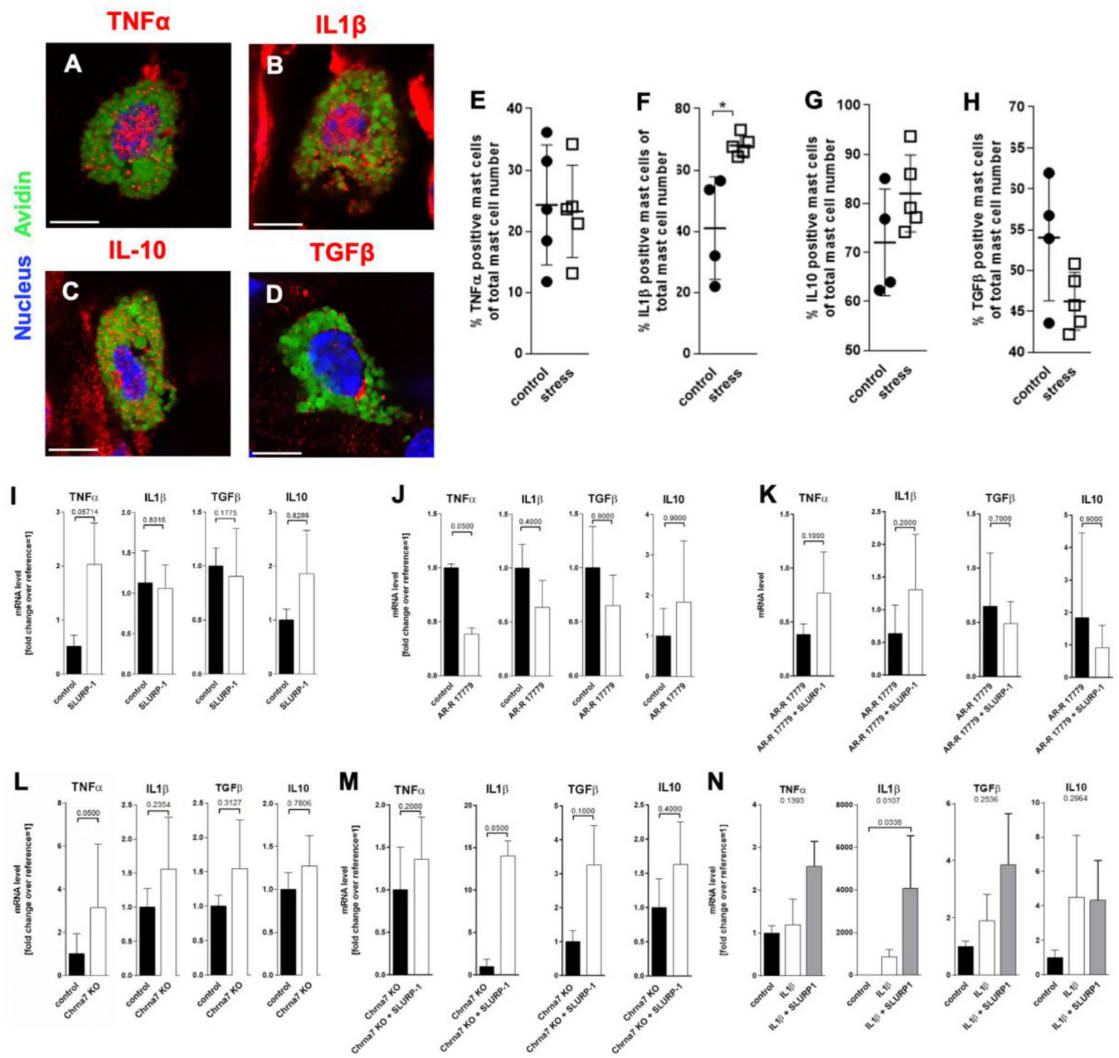
MC cytokine expression is altered in the presence of SLURP-1. **(A–D)** immunohistology and **(E–H)** HM of TNFα, IL1β, IL10, and TGFβ in full-thickness skin samples obtained from 24 h noise-stressed mice. **s**MC are labeled green, cytokines red. Each dot in the graphs represents one mouse. **(I–N)** *N* = 3 WT **c**MC stimulation and co-stimulation with SLURP-1, AR-R 17779 and IL1β and Chrna7-KO stimulation with SLURP-1. Of note, these experiments were done with **c**MC stimulated for 2, 8, 24 as well as 32 h and the clearest results reached after 24 h are shown: SLURP-1 24 h, AR-R 17779 8 h pre-incubation prior to 24 h SLURP-1, IL1β 24 h, and SLURP-1 8 h pre-incubation prior to 24 h IL1β. *P* < 0.01 = *. Scale bar: **(A–D)** 10 μm. Chrna, alpha nicotinic acetylcholine receptor; IL, interleukin; SLURP, Secreted Ly-6/uPAR-related protein; TGF, transforming growth factor; TNF, tumor necrosis factor.

To learn if SLURP-1 and Chrna7 regulate MC's cytokine production after stress exposure in addition to the observed MC degranulation, we next aimed to identify potential target-cytokines by immunohistochemistry in full thickness skin biopsies from control and 24 h noise stressed mice. This revealed prominent IL1β expression in **s**MC 48 h after a 24 h noise stress exposure as well as expression of TNFα-, IL10-, or TGFβ by **s**MC both in control and stressed skin (Figures 4A–D). HM of the cytokine-positive **s**MC revealed a significant increase in the percentage of IL1β- but not TNFα-, IL10-, or TGFβ-immunoreactive **s**MC in stressed mice (Figures 4E–H).

With this in mind, we expected SLURP-1 to enhance IL1β production. However, in WT **c**MC, SLURP-1 had only a mild and non-significant effect on cytokines and upregulated primarily TNFα mRNA (Figure 4I). This contrasted the downregulation of TNFα mRNA by the Chrna7 agonist AR-R 17779, which was comparable to the increase in TNFα mRNA in **c**MC from Chrna7-KO mice and did not occur when AR-R 17779 was given together with SLURP-1. SLURP-1 did thus not act as a Chrna7 ligand but rather as a blocker of Chrna7 downregulation of TNFα (Figures 4J–L). Interestingly, SLURP-1 dramatically upregulated IL1β in **c**MC from Chrna7-KO suggesting that SLURP-1 effects on IL1β are blocked by Chrna7 signaling. However, WT **c**MC stimulation with SLURP-1 in the presence of IL1β resulted in a dramatic upregulation of IL1β indicating that SLURP-1 effects on IL1β are not blocked by Chrna7 signaling under stress-induced inflammatory conditions (Figures 4M,N).

### HIF1α and Oxidative Stress Are Intermediaries Between SLURP-1 and IL1β Upregulation

**Figure 5:**
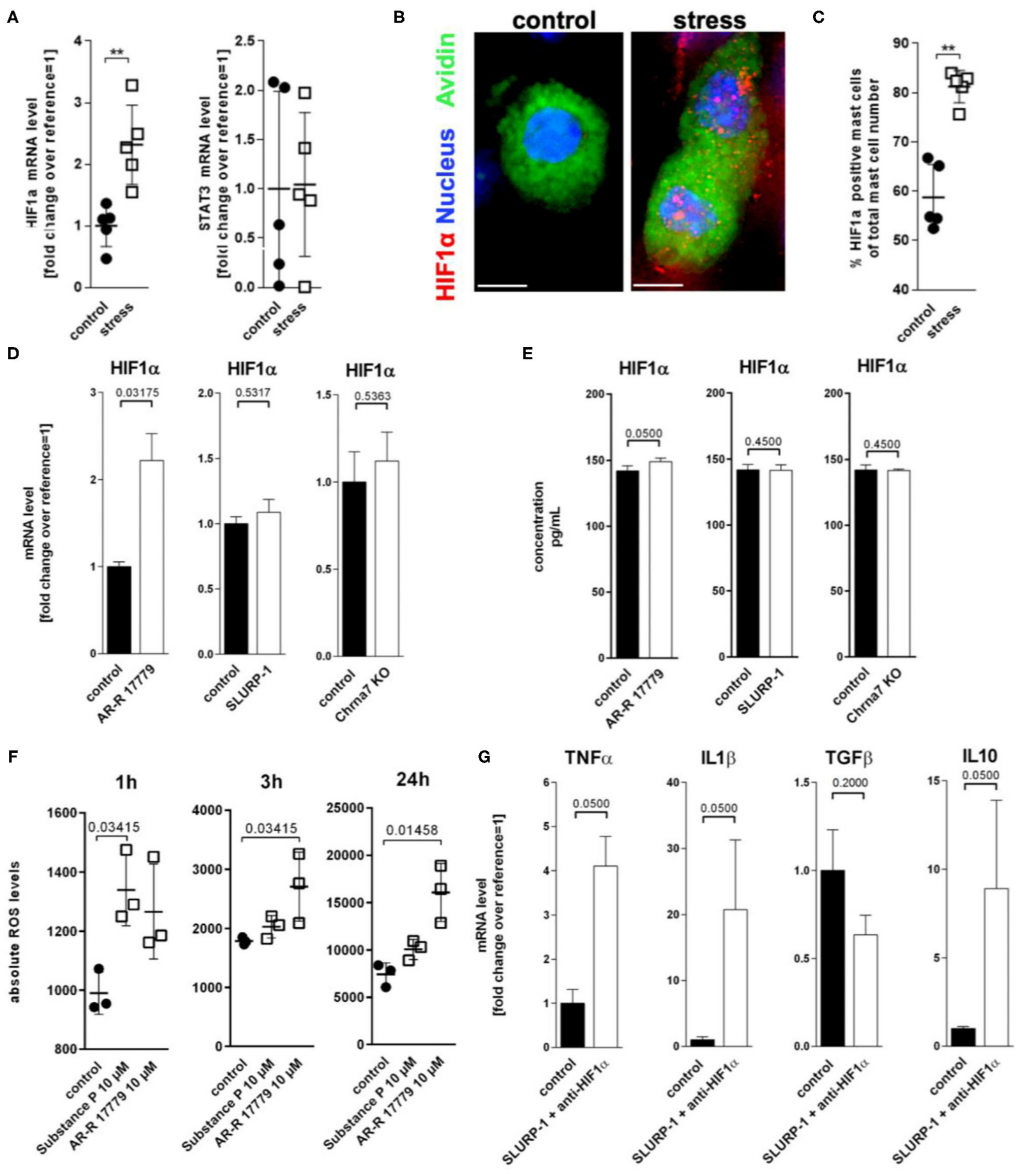
ROS mediated HIF1α upregulation inhibits TNFα production. **(A)** qPCR of HIF1α and STAT3, **(B)** immunohistology, and **(C)** HM of HIF1α in full-thickness skin samples obtained from control and 24 h noise-stressed mice. **s**MC are labeled green, HIF1α red. **(D,E)** *N* = 3 WT **c**MC stimulation with AR-R 17779 increased HIF1α mRNA and protein while treatment with SLURP-1 or Chrna7 KO did not change levels compared with control. **(F)** absolute ROS levels measured after 1, 3, and 24 h. wt **c**MC were stimulated with SP and AR-R 17779. Each dot in **(A,C,F)** represents one mouse. **(G)** *N* = 3 WT **c**MC co-stimulation with SLURP-1 and a HIF1α inhibitor. Results for AR-R 17779 24 h and HIF1α inhibitor 8 h pre-incubation prior to 24 h SLURP-1 are shown. *P* < 0.001 = **. Scale bar: **(B)** 10 μm. HIF, hypoxia inducible factor; ROS, reactive oxygen species; STAT, signal transducer and activator of transcription; SLURP, Secreted Ly-6/uPAR-related protein.

To learn more about intermediaries between SLURP-1 and cytokine regulation in MC in response to stress, we next studied inflammatory transcription factors focussing on two transcription factors potentially regulated by SLURP-1 and Chrna7: HIF1α (77–79) and STAT3 (80). Reassessing our previously published microarray, which compared skin samples derived from control with samples from stressed mice (6), we found that HIF1α was upregulated in stressed skin compared to control (fold change 0.414 and STAT3 was downregulated (fold change −0.996). qPCR of full thickness skin samples confirmed this observation for HIF1α but revealed stable levels for STAT3 (Figure 5A). To assess if MC were the source of HIF1α production, we again employed HM and found HIF1α expression in **s**MC in a granular pattern (Figure 5B) mostly at the dermis-subcutis border. We also found higher numbers of HIF1α + **s**MC in stressed mice (Figure 5C).

To test the potential CS regulation of HIF1α transcription, we employed **c**MC and found that only the Chrna7 agonist AR-R 17779 but not SLURP-1 or Chrna7-KO upregulated HIF1α mRNA and protein (Figures 5D,E). Since it is known that HIF1α activity can be enhanced by ROS (81), that stress increases ROS (82), that and H_2_O_2_ regulates nicotine-induced HIF1α expression via Chrna7-mediated signaling pathways (78), we wanted to know whether ROS are involved in the observed regulation. We therefore used a DCF-DA assay to measure intracellular H_2_O_2_ and found that AR-R 17779 had a lasting stimulatory effect on ROS production in **c**MC. We also confirmed the reported SP upregulation of ROS (Figure 5F). However, this effect was rather short lived. To finally learn if the HIF1α induction by Chrna7 interacts with SLURP-1 effects, we studied SLURP-1 effects in **c**MC after HIF1α blockade. This approach led to a significant TNFα and IL1β increase by SLURP-1 (Figure 5G).

Finally, to assess whether the observed upregulations relate to each other, **s**MC HM and mRNA data were submitted to a Spearman's rank correlation. We found that upregulation of CS components Chrna7 and SLURP-1, as well as of IL1β and HIF1α significantly correlated with each other (Table 6). These findings support a strong connection of these components in stress.

**Table 6:** Spearman's rank correlation between sMC HM and mRNA data.

	**A**	**B**	**C**	**D**	**E**	**F**	**G**	**H**
A: % Chrna7+ of total MC number (*N* = 5)								
B: % MC with SLUPR-1 pos. nerve cont. (*N* = 3)	0.900							
C: % IL1β+ MC of total MC number (*N* = 4)	0.833*	0.943*						
D: % HIF1α + of total MC number (*N* = 5)	0.883**	0.771	0.917**					
E: Chrna7 1-4 mRNA level (*N* = 5)	0.583	0.657	0.650	0.697*				
F: Chrna7 5-7 mRNA level (*N* = 5)	0.650	0.657	0.700*	0.697*	0.806**			
G: Chrna7 8-10 mRNA level (*N* = 5)	0.733*	0.886*	0.683	0.721*	0.794**	0.794**		
H: SLURP-1 mRNA level (*N* = 5)	0.767*	0.771	0.833**	0.806**	0.672*	0.818**	0.660*	
I: HIF1α mRNA level (*N* = 5)	0.800*	0.828	0.717*	0.721*	0.588	0.830**	0.806**	0.830**

Spearman's rank correlation coefficient rho is shown. Rho ranges between −1 and +1. The closer rho is to −1 or +1, the stronger the correlation. A perfect negative Spearman correlation is −1, a perfect positive correlation is +1. Statistically significant values are printed in bold and denotes the strength of the correlation. P < 0.05 = *. P < 0.001 = **. Chrna, alpha nicotinic acetylcholine receptor; HIF, hypoxia inducible factor; IL, interleukin; MC, mast cells; SLURP, secreted Ly-6/uPAR-related protein.

## Discussion

The here present experiments aim to clarify the CS involvement in inflammatory stress responses of the healthy organism in an established experimental stress paradigm. Our results first revealed a highly selective upregulation of individual components of the CS in healthy mouse skin in response to a 24 h noise stress exposure, namely Chrna7 and its peptidergic modulator SLURP-1. With respect to neuro-immune communication, immunohistochemistry located these two markers primarily to **s**MC and nerve fibers that were located close by the **s**MC in the skin of stressed mice. This suggested a role of their interaction in neurogenic inflammation, as was previously described for sensory neuropeptides such as SP (36). MC were next confirmed as relevant cellular targets of the skin's CS stress response because SLURP-1 was able to degranulate **c**MC. In addition, it promoted **c**MC cytokine production in favor of an innate pro-inflammatory state. We hence show for the first time that SLURP-1 is a MC degranulating peptide and that it is used by the CS to contribute to stress-MC activation. The thus supported pro-inflammatory state is well-suited to contribute to efficient host defense and maintain homeostasis in healthy skin.

That the CS does not play its expected anti-inflammatory role in stressed healthy skin is intriguing for a number of reasons and requires in depth discussion, as it contrasts the anti-inflammatory cholinergic axis concept. This can have widespread implications for skin pathologies ranging from allergies via infectious diseases to tumor development (9, 33, 36).

For one, the previously published suggestive evidence for an anti-inflammatory effect of SLURP-1 in skin disease and allergy was strong. Individuals with a mutation that disrupts SLURP-1 function suffer from Mal de Meleda, a transgressive and progressive palmoplantar inflammatory disease resembling the effects of MC hyperactivation in atopic dermatitis (83, 84). Also, SLURP-1 is often considered to be an allosteric agonist of Chrna7 and such agonists were shown to inhibit MC degranulation (20). Moreover, SLURP-1 was detected in skin nerve fibers shortly after its first description in 1999 (44, 85, 86) and close contacts of calcitonine gene related peptide+ cholinergic nerve fibers with Chrna7+ MC were reported in a food allergy model, in which Chrna7 agonists attenuated MC hyperplasia and allergic inflammation (87).

For another, our results contrast a number of studies that clearly state an anti-inflammatory and hence classical Chrna7 agonistic effect of SLURP-1 in other tissues. Recombinant SLURP-1 decreased for example the TLR9-dependent secretion of IL-8 by colonocytes, the IFNγ-induced upregulation of ICAM-1 in enterocytes, the production of TNFα by T-cells, and the secretion of IL1β and IL6 by macrophages (88). These results supported the longtime accepted assumption that SLURP-1 is an allosteric agonist of Chrna7 and promotes an anti-inflammatory state.

Our study, however, supports the contrasting concept that close contacts of SLURP-1+ nerve fibers with **s**MC provide a neurogenic circuit in stressed skin that can boost inflammatory processes by leading to MC degranulation and pro-inflammatory cytokine production (7, 16). It is possible that these pro-inflammatory effects of SLURP-1 are due to its inhibition of Chrna7 signaling or the desensitization of Chrna7 to its agonists (89–93). This concept is supported by the report that the exposure of Chrna7+ Xenopus oocytes to recombinant SLURP-1 leads to a non-competitive inhibition of the response to acetylcholine (94). Moreover, SLURP-1 was found to antagonize biological functions attributed to Chrna7 in patients with pancreatic cancer (95) or malignant melanoma (96). In skin plagued by an inflammatory disease this pro-inflammatory effect could hence have deleterious effects and worsen inflammation, while it could protect from cancer progression and potentially viral infections in other contexts (33, 41, 97).

Notably, MC can produce and release cytokines selectively and acute stress can promote the transient production and release of these pro-inflammatory cytokines (98). Cytokines that regulate innate and adaptive immune responses such as IL1β or TNFα are thereby *de novo* synthesized and released independently from degranulation-associated signaling pathways (99), as shown for example in the late phase of MC activation induced by the high-affinity receptor for IgE (Fc epsilon RI) (20, 21).

That we found high IL1β 48 h after noise stress and that SLURP-1 enhanced IL1β in **c**MC under certain conditions, while Chrna7 was unable to downregulate TNFα in combination with SLURP-1, is intriguing. In the pathophysiology of inflammatory diseases Chrna7-mediated downregulation of TNFα is widely studied and its respective anti-inflammatory benefits are appreciated in a number of highly acute inflammatory disease states, but clinical effects of Chrna7 targeting treatments do not always bring the expected beneficial results (89–93). Our results imply that it is important to control for SLURP-1 in respective settings. Also, further studies are required to address the interaction of SLURP-1 with other Chrna.

Current studies suggest that SLURP-1 can be a ligand of Chrna9 (100), which was shown to be upregulated in the absence of Chrna7 (101, 102). Interestingly, the specific Chrna7 antagonist MG624 was lately shown to also act on Chrna9 containing hetero-pentamers in cancer cells (103). Since Chrna9 was shown to be involved in the inhibition of the release of IL1β in response to the danger signal extracellular ATP (104), the here reported IL1β upregulation in Chrna7-KO mast cells after stimulation with SLURP-1 could be mediated by SLURP-1 interference with Chrna9.

Overall, it would be interesting to learn, what role plays SLURP-1 in the NLRP3 inflammasome induced release of IL1β as this was recently shown to be present in MC (105) and to be attenuated by classical Chrna7 activation in a stroke model (106). Regulation of NLRP3 inflammasome activation could represent a target for future pharmacologic interventions employing SLURP-1. This is especially interesting in chronic inflammatory skin and allergic diseases (107) that reportedly are stress-sensitive and involve NLRP3 inflammasome activation such as psoriasis (108), contact hypersensitivity (109) or asthma (110). In fact, in a microarray analysis employing the Affymetrix Mouse Genome 430A 2.0 Array that we had performed in another project (6), we had previously observed the as yet unreported upregulation of Asc (1.280-fold) and Casp1 (0.397-fold). Since IL1β can boost inflammatory diseases associated with IL1β overexpression (111–113) the possibility to regulate IL1β levels via SLURP-1 is intriguing.

Concerning the studied transcription factors targeted by Chrna7, the stress did not regulate STAT3 but HIF1α. This provides additional information on the CS regulation of stress responses. HIF1α was shown to be upregulated in MC when cells mature or locate near a bacterial infection (114–116). Also, HIF1α was upregulated simultaneously with MC infiltration in nasal epithelium of cigarette smoke exposed mice (117). This protects from infection, as MC exhibit increased microbicidal activity and more efficient control of invasive bacterial infection, when HIF1α is increased (118). At the same time, oxidative stress and HIF1α reduce TNFα in MC through Chrna7 signaling (78, 81, 119). Hence, through HIF1α upregulation, the CS efficiently protects from bacterial infection by boosting innate immunity and hampering the switch to adaptive immunity.

Moreover, HIF1α upregulation together with stable or downregulated STAT3 may have implications for tumor growth as HIFα can hamper tumor growth. In a number of human and murine MC HIF1α was shown to be upregulated when cells mature or locate near a tumor (114–116) and in meningeomas HIF1α immunoreactivity correlates with occurrence of tryptase+ MC (120) suggesting a role for MC in the HIF1α mediated defense against tumor growth. In addition, SLURP-1 was shown to abolish STAT3 upregulation in cancer cells (121).

Of course, our study has limitations. We used a purchasable recombinant SLURP-1 protein with a GST-tag at the N-terminal. As recently reported, N-terminal extensions could affect the activity and selectivity of SLURP-1 on its targets (100). Also, it cannot be excluded that SLURP-1 effects could be tissue-, cell- or dose-dependent and could depend on the nature of inflammatory stimuli and, respectively, the corresponding signaling pathways. As, immunohistochemically, SLURP-1 and Chrna7 expression was most prominent on **s**MC and nerve fibers, the present study focused on both as important players in the skin's CS stress response. Future studies should analyse the involvement of additional immune cells. Finally, the clinical relevance of our observations has to be tested in appropriate disease models, which is presently under way.

## Conclusion

**Figure 6:**
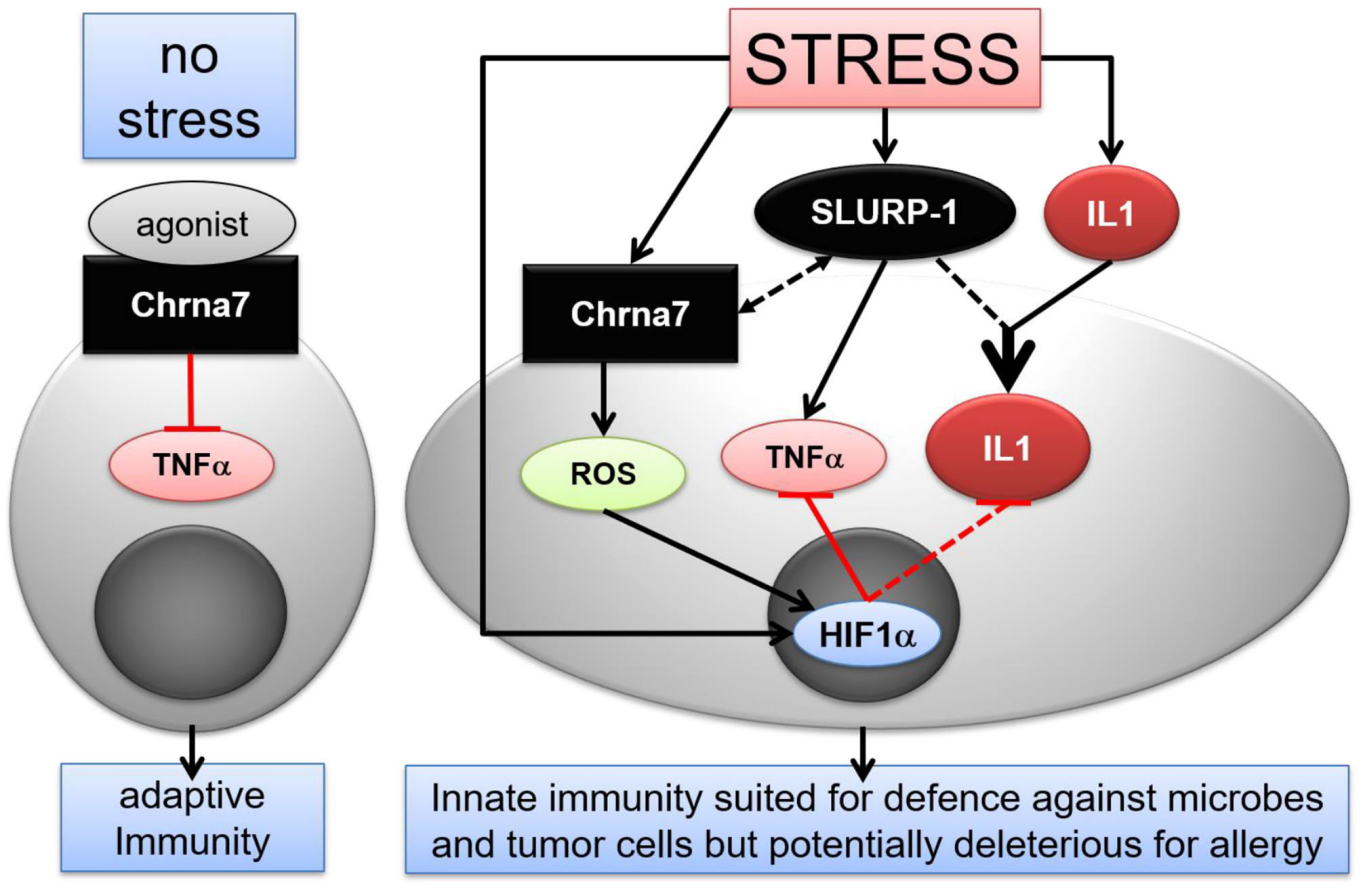
Hypothetical scenario of stress interference with CS mediated immune modulatory effects in murine skin MC. Under normal conditions, activation of Chrna7 inhibits TNFα production by MC. In stress, however, SLURP-1 induces a shift in the immune response toward an innate pro-inflammatory state. SLURP-1 upregulates TNFα and IL1β expression by MC. Together with SLURP-1 and Chrna7, IL1β, and HIF1α are upregulated in MC by stress. Thereby, SLURP-1 promoted TNFα and IL1β production is inhibited by Chrna7 and ROS mediated HIF1α upregulation. However, SLURP-1 and IL1β synergize to produce an innate pro-inflammatory state. Thereby SLURP-1 may act as an allosteric ligand to Chrna7 and through Chrna7-unrelated, yet to be identified receptors (45, 88). Chrna, alpha nicotinic acetylcholine receptor; HIF, hypoxia inducible factor; IL, interleukin; ROS, reactive oxygen species; SLURP, Secreted Ly-6/uPAR-related protein; TNF, tumor necrosis factor.

Taken together, our findings suggest that in stressed healthy skin, SLURP-1 induces a shift in the immune response toward a pro-inflammatory state and thereby enables the skin's CS to orchestrate an efficient defense against microbes and tumor cells, a response that aims at the maintenance of homeostasis (94). At the same time, this potentially puts the skin at risk for deleterious stress effects if hit by additional challenges. This could for example contribute to the stress-induced worsening of allergic inflammation that we had observed earlier (4). Evidently, the CS elements that were upregulated 24 h noise stress in healthy skin did not directly interact to activate MC and produce a pro-inflammatory cytokine profile. Instead, they teamed up to prepare the skin for an innate immune response (Figure 6) (122). Further investigations will show the relevance of these results for the control of infectious diseases and cancer and for the treatment of non-infectious inflammatory disorders such as atopic dermatitis. This is presently tested in our laboratory in corresponding mouse models.

## Data Availability Statement

The original contributions presented in the study are included in the article/supplementary materials, further inquiries can be directed to the corresponding author/s.

## Ethics Statement

The animal study was reviewed and approved by Landesamt für Arbeitsschutz, Gesundheitsschutz und technische Sicherheit, Berlin, Germany.

## Author Contributions

EP and UG designed the study. EP and CE drafted the manuscript. CE and FR conceived and carried out experiments. EP, YM, and JKl conceived experiments and analyzed data. ST carried out experiments. FR, YM, JKl, and JKr critically reviewed, edited, and commented the initial draft. All authors were involved in writing the paper and had final approval of the submitted and published versions.

## Conflict of Interest

The authors declare that the research was conducted in the absence of any commercial or financial relationships that could be construed as a potential conflict of interest.

## Abbreviations

Ach: Acetylcholine
AChE: acetylcholinesterase
AID: atopic-like dermatitis allergic dermatitis
BChE: butyrylcholinesterase
Ch: choline
ChAT: choline acetyltransferase
Chrm3: muscarinic acetylcholine receptor 3
Chrna7: alpha nicotinic acetylcholine receptor 7
Chrna7-KO: alpha nicotinic acetylcholine receptor 7 knockout
Chrna9: alpha nicotinic acetylcholine receptor 9
cMC: cultured mast cells
CS: cholinergic system
DCFH-DA: dichlorofluorescein diacetate
DNP-HAS: dinitrophenyl albumin
HAD: β-hexosaminidase degranulation assay
HM: histomorphometry
HIF1α: hypoxia inducible factor 1 alpha
HPLC: high-performance liquid chromatography
IHC: immunohistochemistry
IL10: interleukin 10
IL1β: interleukin 1 beta
Lynx1: Ly6/Neurotoxin 1
MC: mast cells
NGF: Nerve Growth Factor
Oct3: organic cation transporter 3
PMA: Phorbol 12-myristate 13-acetate
ROS: reactive oxygen species
SLURP-1: secreted Ly-6/uPAR-related protein 1
SLURP-2: secreted Ly-6/uPAR-related protein 2
sMC: skin mast cells
SP: substance P
STAT3: signal transducer and activator of transcription 3
TBP: TATA box binding protein
TGFβ: transforming growth factor beta
TNFα: tumor necrosis factor alpha
VAChT: vesicular acetylcholine transporter
WT: wildtype.
